# Supplementary material for: Metabolic engineering of Escherichia coli for de novo biosynthesis of vitamin B12
Source: Nat Commun. 2018 Nov 21;9:4917. doi: 10.1038/s41467-018-07412-6 (PMC6249242; doi:10.1038/s41467-018-07412-6)
Supplement: Supplementary file 1 — Supplementary Information [file 41467_2018_7412_MOESM1_ESM.pdf]

Metabolic engineering of *Escherichia coli* for *de novo* biosynthesis of vitamin B_{12}

Fang et al.

Supplementary Note 1

Consider that the efficiencies of the CobN, CobS, and CobT enzymes from different bacteria are all different and that our SDS-PAGE analysis (Supplementary Fig. 7) showed that each was expressed in our *E. coli* strain at different levels. We therefore examined a total of 9 combinations of CobN, CobS, and CobT proteins from different bacteria. CBAD was formed in all combinations when the substrate HBAD, the three enzymes (CobN, CobS, and CobT), and $CoCl_2$ were all present, but was not detected in the cotrol group lacking $CoCl_2$ (Supplementary Figs. 5, 6).

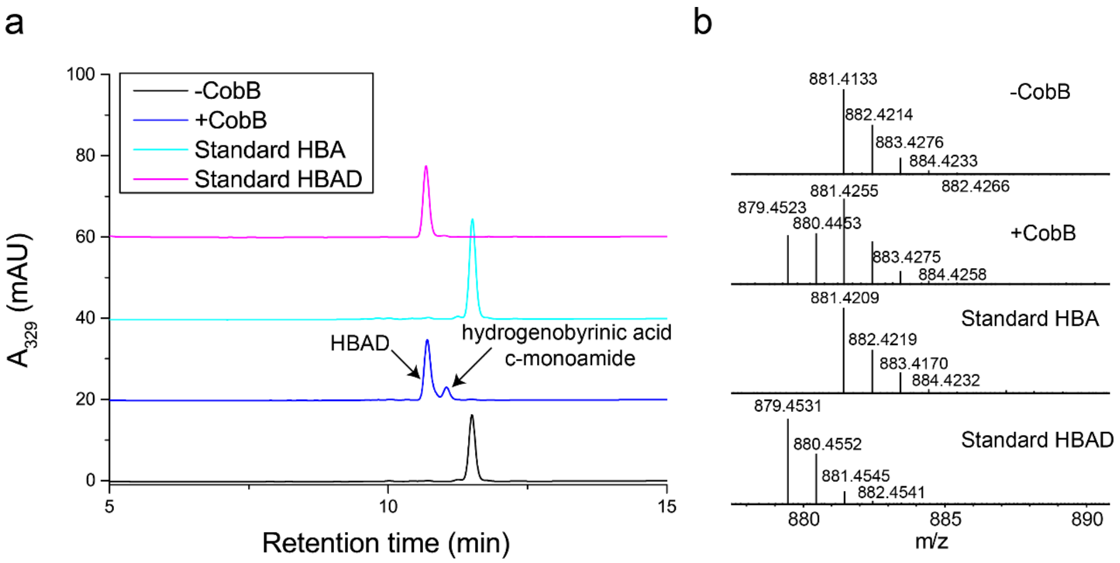

Supplementary Figure 1. Verifying the function of CobB from *Rhodobacter capsulatus* by LC-MS. **(a)** HPLC analysis of the in vitro assay product of CobB from *R. capsulatus*. CobB was excluded in the sample shown in black but was included in the sample shown in blue. **(b)** MS analysis of HBAD formed by CobB using HBA as the precursor.

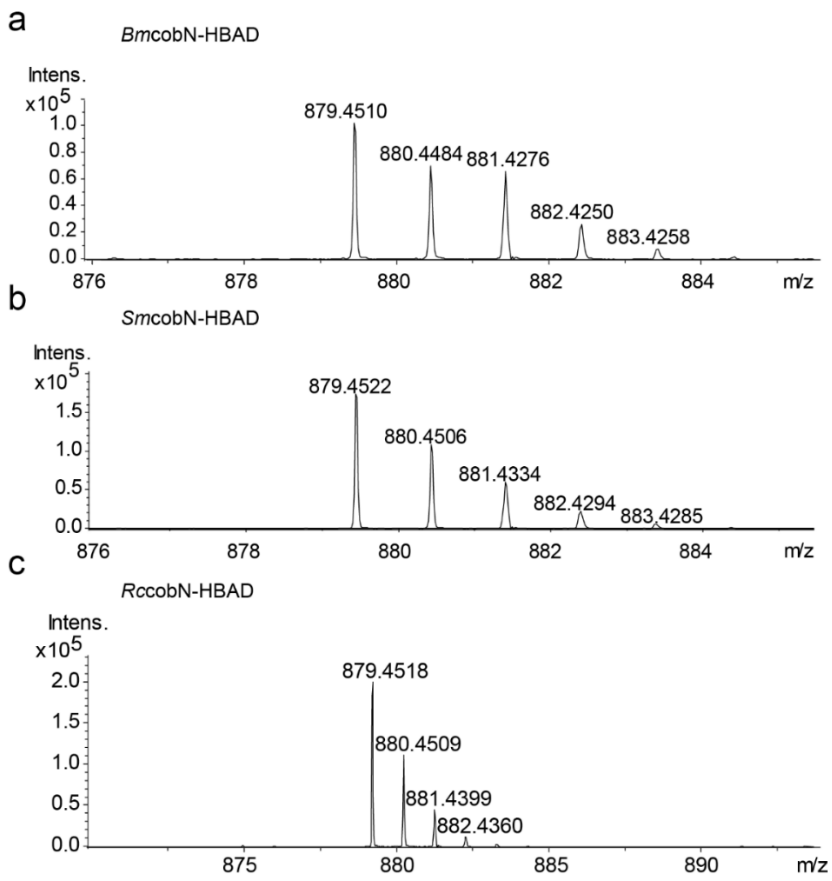

Supplementary Figure 2. LC-MS analysis of HBAD bound to CobN from *B. melitensis*, *S. meliloti*, and *R. capsulatus*. HBAD was obtained by purification of CobN from *Brucella melitensis* (**a**), *Sinorhizobium meliloti* 320 (**b**), and *R. capsulatus* (**c**) via a modified enzyme-trap method[1] for analysis.

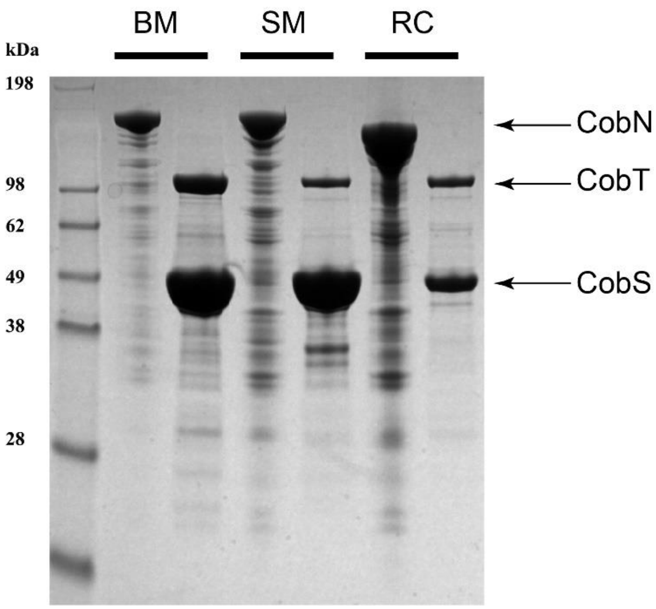

Supplementary Figure 3. SDS-PAGE analysis of purified CobN, CobS, and CobT from *B. melitensis*, *S. meliloti*, and *R. capsulatus*. CobN was expressed separately via the the plasmids pCDF-cobB-BmcobN-his, pCDF-cobB-SmcobN-his, and pCDF-cobB-RccobN-his, respectively. CobS and CobT were expressed on the same plasmid pACYCDuet-1 for copurification. CobN, CobS, and CobT were purified by affinity chromatography using a Ni Sepharose column.

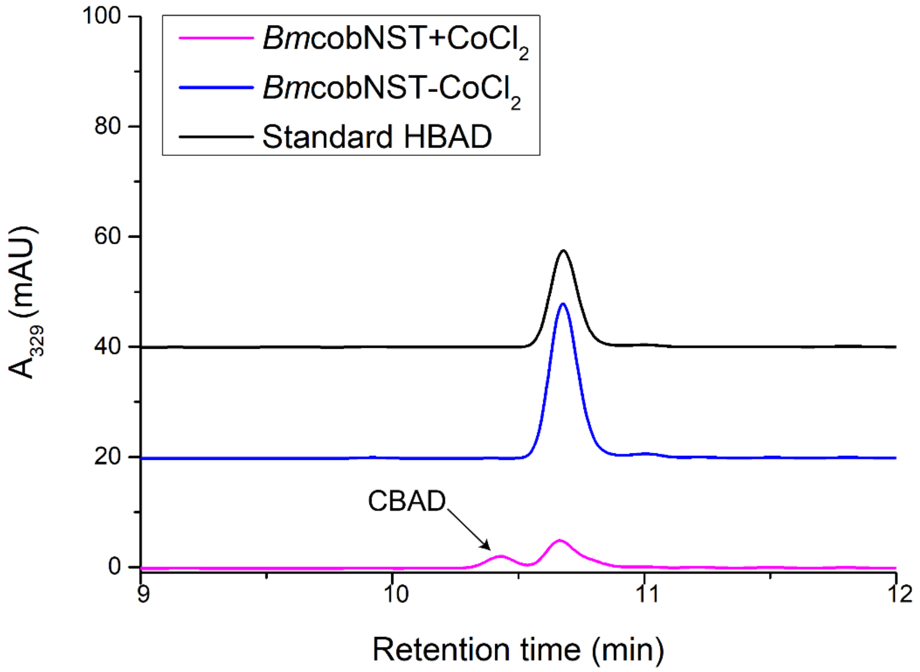

Supplementary Figure 4. HPLC analysis of the in vitro assay product of the CobN, CobS, and CobT enzyme reaction from *B. melitensis*. The control sample excluded $CoCl_2$ from the reaction mixture.

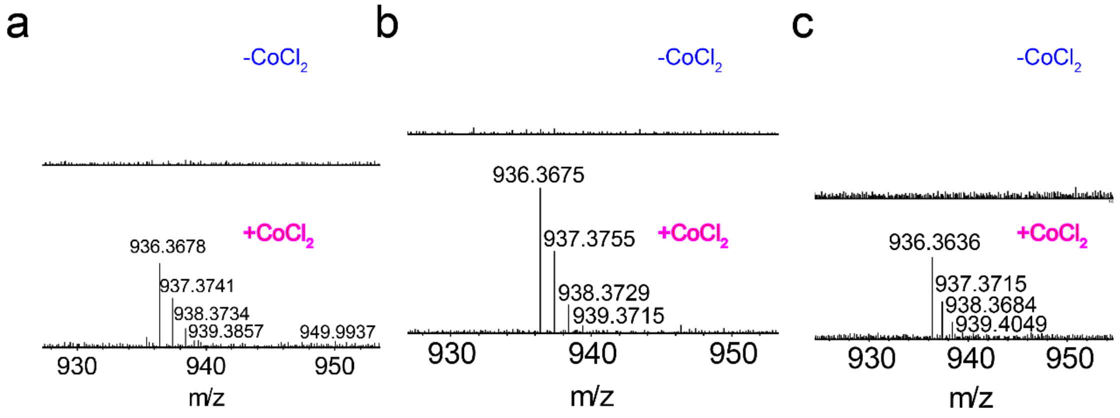

Supplementary Figure 5. LC-MS analysis of in vitro assay products formed by CobNST from *B. melitensis* (**a**), *S. meliloti* (**b**), or *R. capsulatus* (**c**). CobN-HBAD, CobST, $CoCl_2$, and other ingredients were included in the experimental samples, while $CoCl_2$ was excluded from the control.

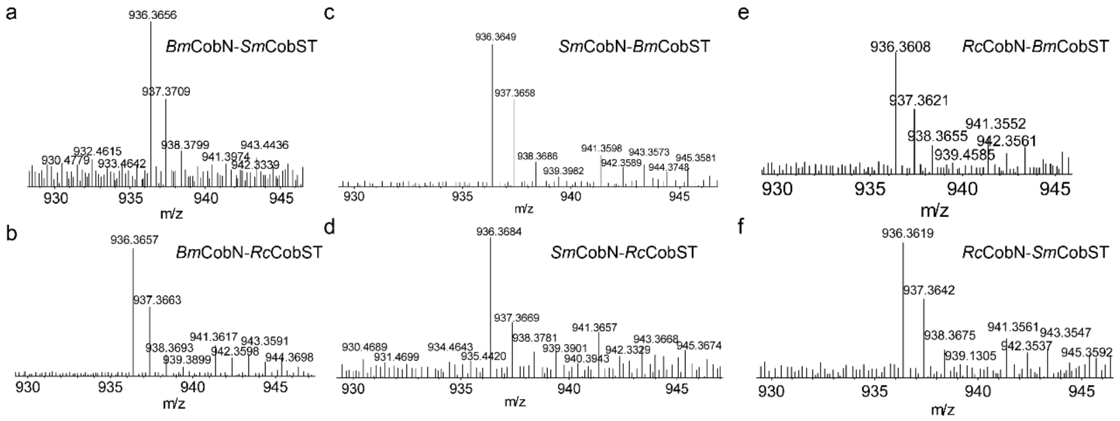

Supplementary Figure 6. LC-MS analysis of products fromed by in vitro reactions that variously combined the CobN, CobS, and CobT enzymes. Specifically, CobN from *B. melitensis* and CobS and CobT from *S. meliloti* (**a**), CobN from *B. melitensis* and CobS and CobT from *R. capsulatus* (**b**), CobN from *S. meliloti* and CobS and CobT from *B. melitensis* (**c**), CobN from *S. meliloti* and CobS and CobT from *R. capsulatus* (**d**), CobN from *R. capsulatus* and CobS and CobT from *B. melitensis* (**e**), CobN from *R. capsulatus* and CobS and CobT from *S. meliloti* (**f**) were tested in vitro for their production of CBAD.

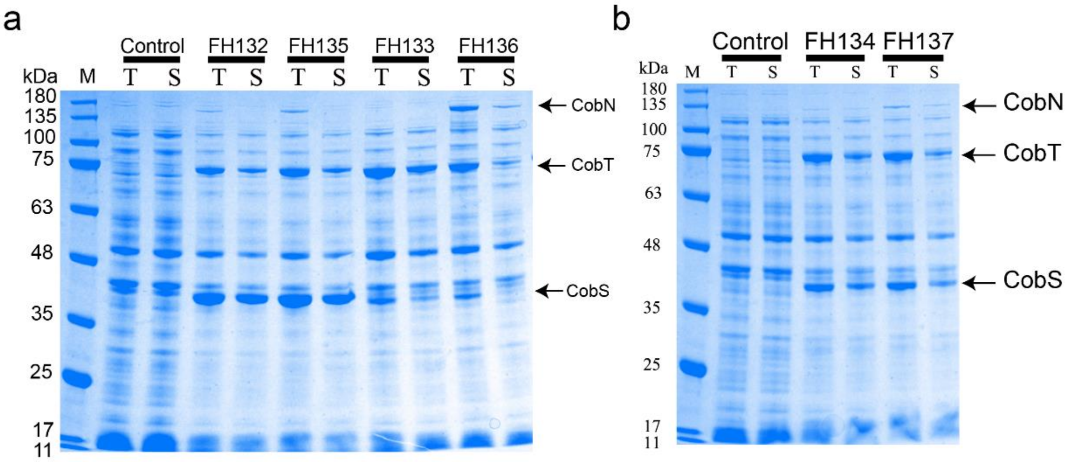

Supplementary Figure 7. SDS-PAGE analysis of the expression of CobN, CobS, and CobT from *S. meliloti*, *B. melitensis*, or *R. capsulatus*. **(a)** Expression of CobN, CobS, and CobT from *S. meliloti* and *B. melitensis* expressed via pCDF-cobB-SmcobN-SmcobS-SmcobT, pCDF-cobB-SmcobN-his-SmcobS-SmcobT, pCDF-cobB-BmcobN-BmcobS-BmcobT, and pCDF-cobB-BmcobN-his-BmcobS-BmcobT. **(b)** Expression of CobN, CobS, and CobT from *R. capsulatus* expressed via pCDF-cobB-RccobN-RccobS-RccobT and pCDF-cobB-RccobN-his-RccobS-RccobT. The *cobN* genes in the FH135, FH136, and FH137 strains were fused with an N-terminal hexa-histidine tag.

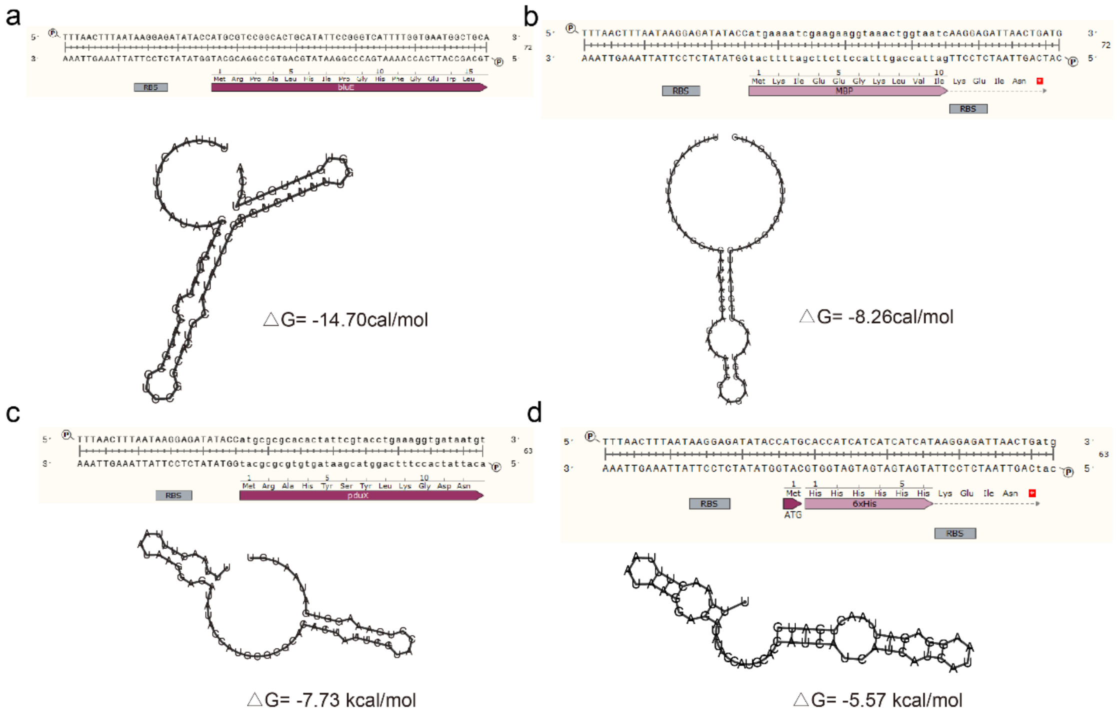

Supplementary Figure 8. Design of bicistronic vectors to increase the translation of BluE and PduX. The mRNA centroid secondary structures with the corresponding minimum free energy (ΔG) for native and engineered sequences were predicted by the RNAfold program. Sequences and secondary structures of original **(a)** and engineered 5´ UTR of *bluE* **(b)**. Sequences and secondary structures of original **(c)** and engineered 5´ UTR of *pduX* **(d)**.

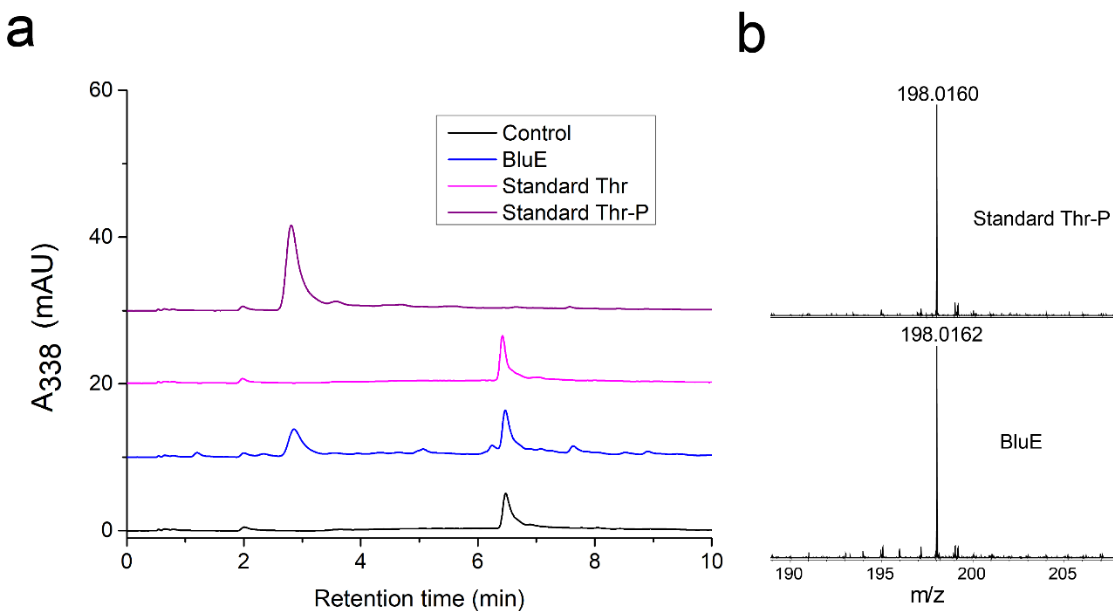

Supplementary Figure 9. Confirmation of the function of BluE in vitro by LC-MS. **(a)** HPLC analysis of the in vitro assay product of BluE from *R. capsulatus*. BluE was excluded from the control group. Thr and Thr-P represent standards for L-threonine and threonine-O-3-phosphate, respectively. **(b)** MS analysis of Thr-P formed by BluE using Thr as the precursor.

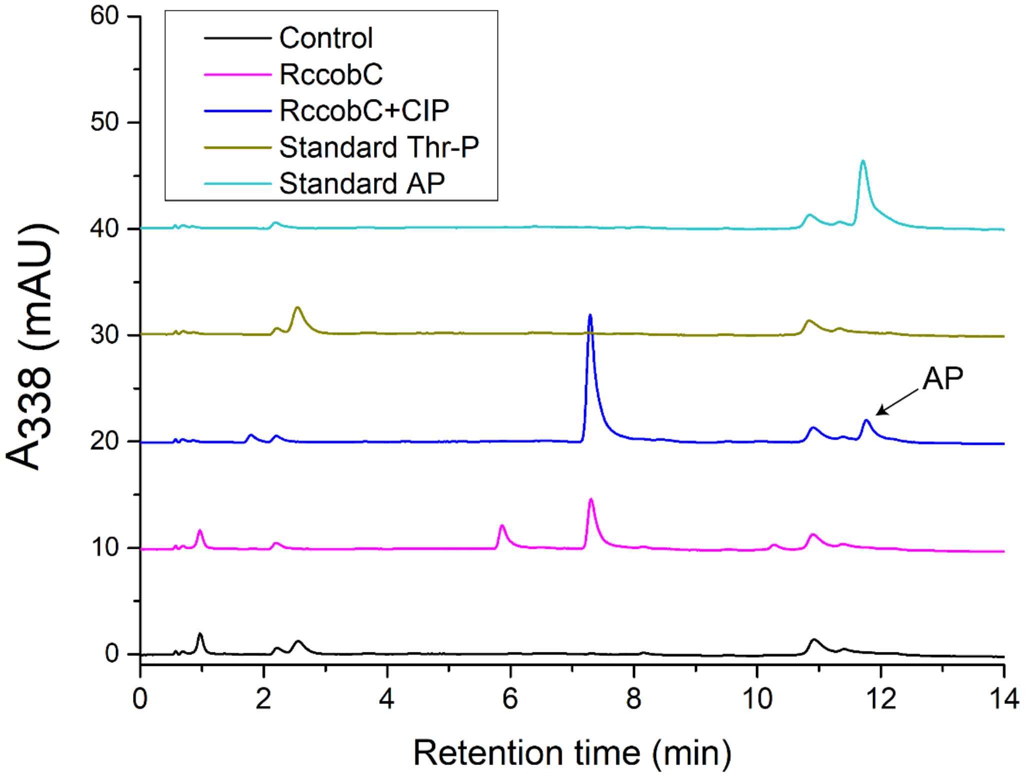

Supplementary Figure 10. Confirmation of the function of *Rc*CobC in vitro. *Rc*CobC was excluded from the control group. APP was converted to AP by CIP (blue line). Thr-P, APP, and AP represent threonine-O-3-phosphate, (R)-1-Amino-2-propanol O-2-Phosphate, and (R)-1-Amino-2-propanol, respectively.

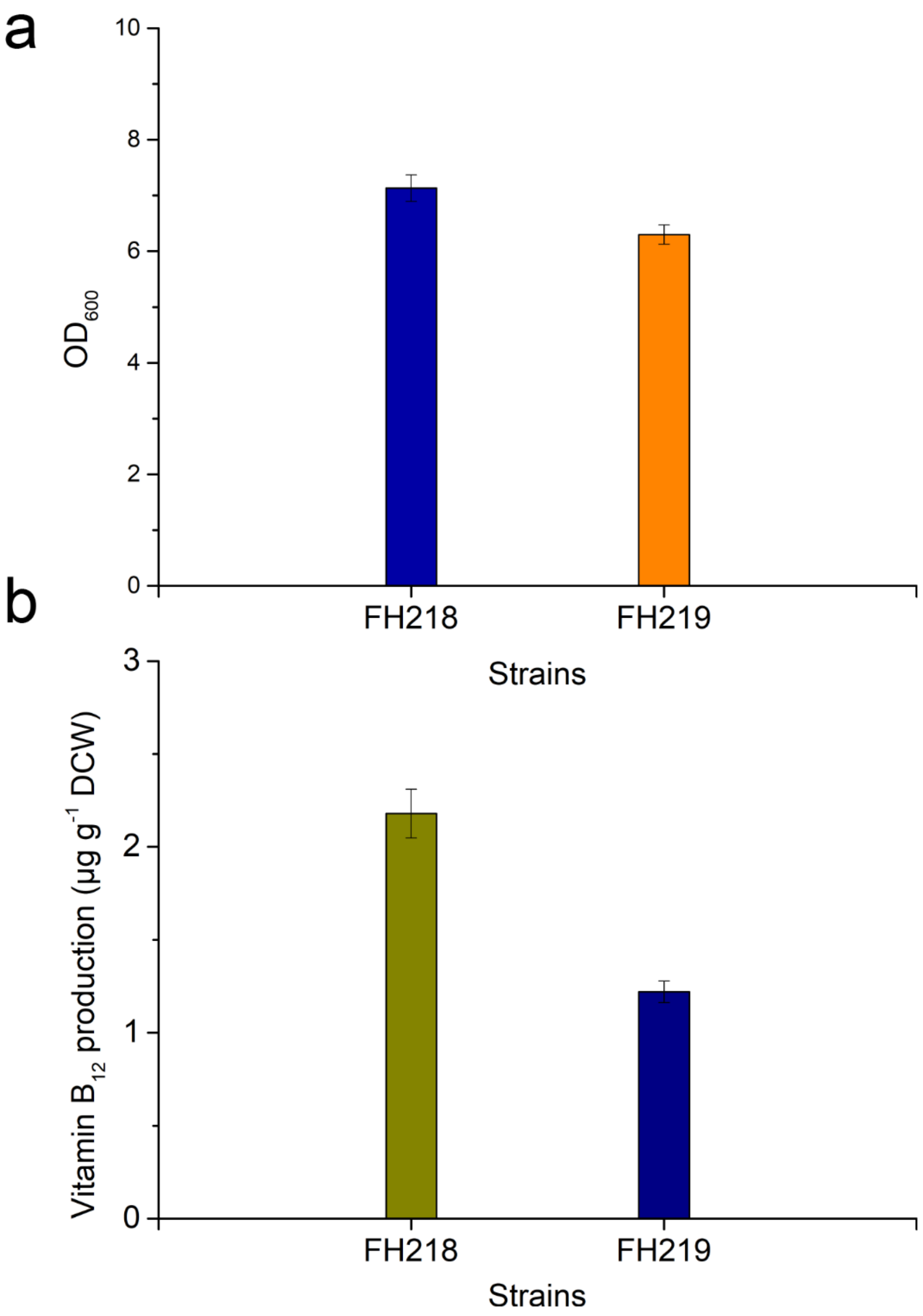

Supplementary Figure 11. Comparison of cell growth (**a**) and vitamin B_{12} production (**b**) of the FH218 and FH219 strains. Both strains were grown in CM medium for vitamin B_{12} production. Error bars indicate standard deviations from triplicate biological replicates.

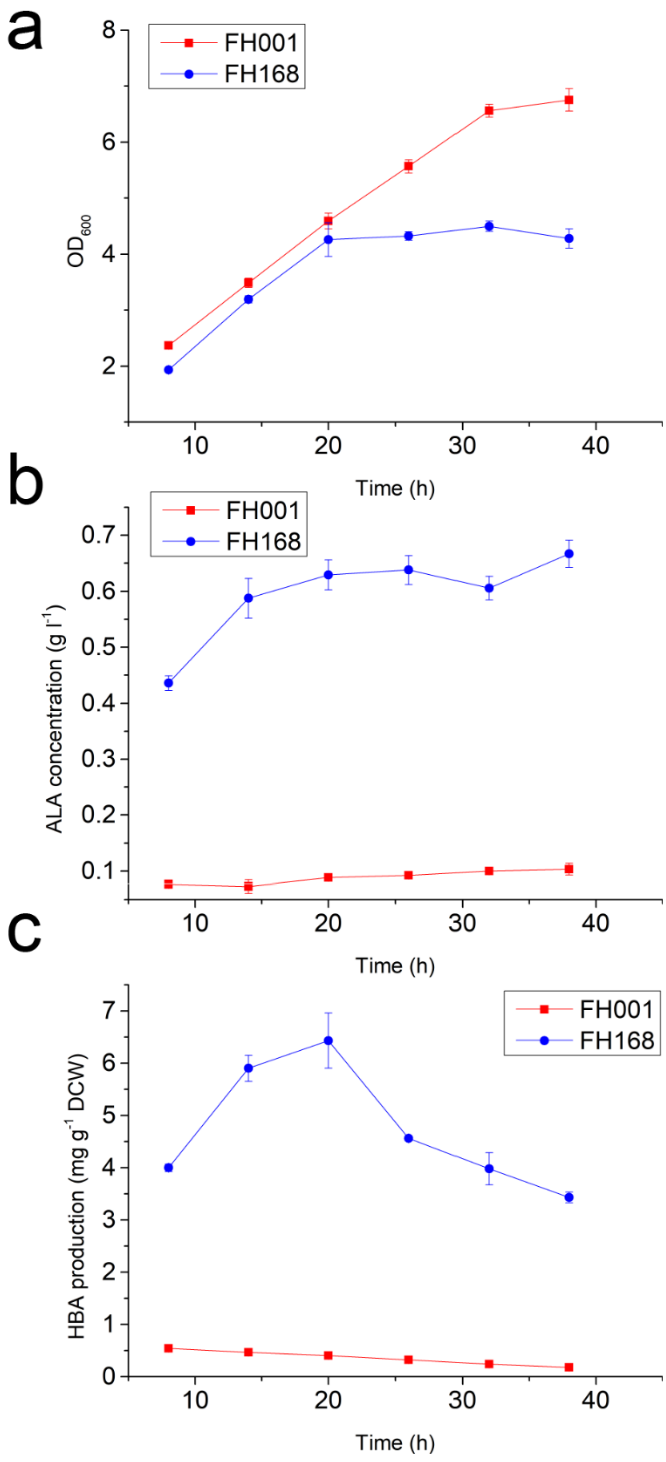

Supplementary Figure 12. Comparison of the (**a**) growth characteristics, (**b**) ALA, and (**c**) HBA production of recombinant strains. FH168 harbors heterologous *hemO*, *hemB*, *hemC*, and *hemD*, while FH001 does not. Both strains were grown in TYG medium for HBA production. Error bars indicate standard deviations from triplicate biological replicates.

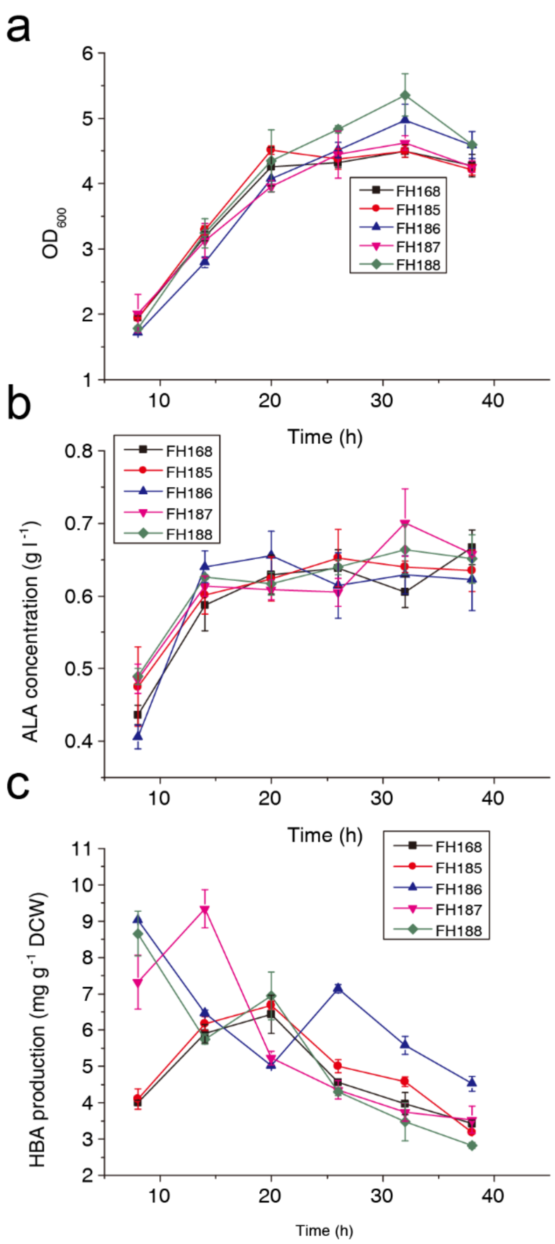

Supplementary Figure 13. Comparison of (**a**) growth characteristics, (**b**) ALA, and (**c**) HBA production of recombinant strains with single gene knockdown. Strains were grown in TYG medium for HBA production. Error bars indicate standard deviations from triplicate biological replicates.

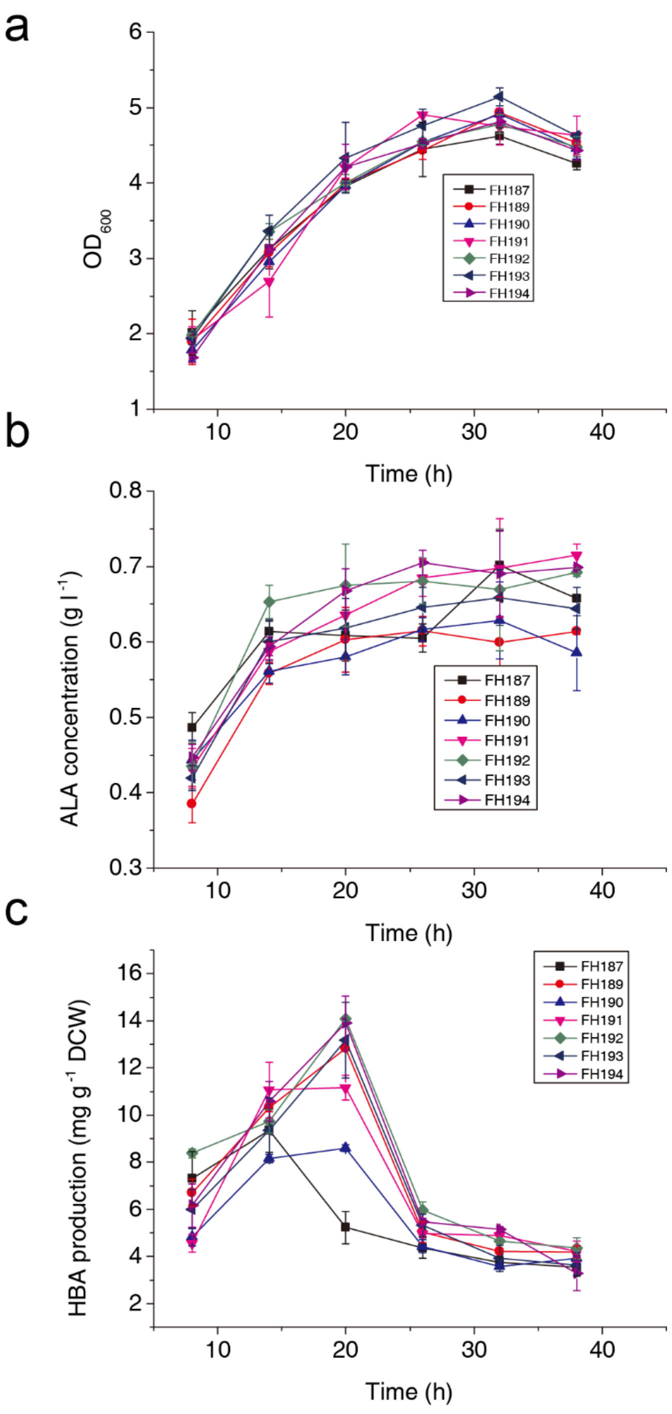

Supplementary Figure 14. Comparison of (**a**) growth characteristics, (**b**) ALA, and (**c**) HBA production of recombinant strains with simultaneous knockdown of two genes. Strains were grown in TYG medium for HBA production. Error bars indicate standard deviations from triplicate biological replicates.

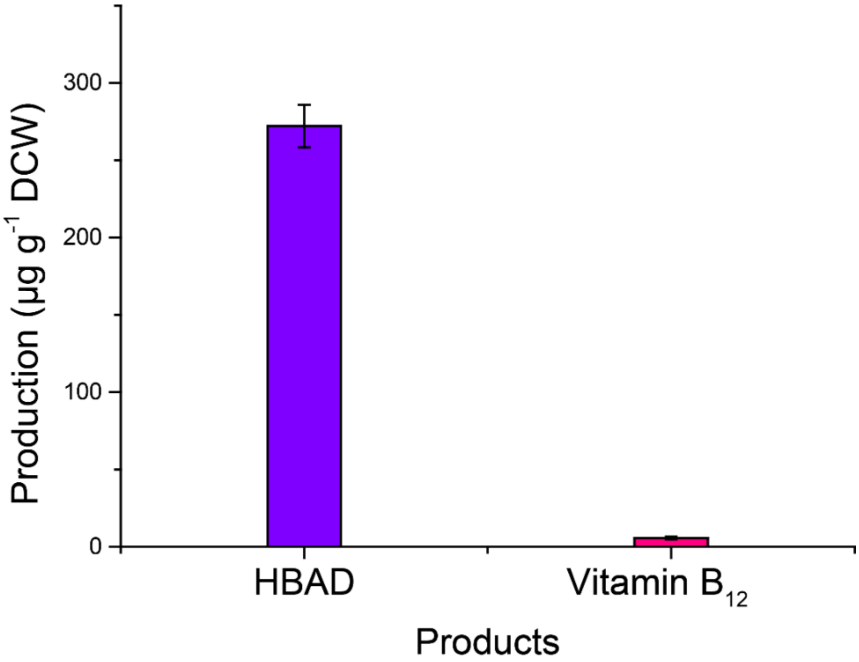

Supplementary Figure 15. Comparison of HBAD and vitamin B_{12} production of FH309. Error bars indicate standard deviations from triplicate biological replicates.

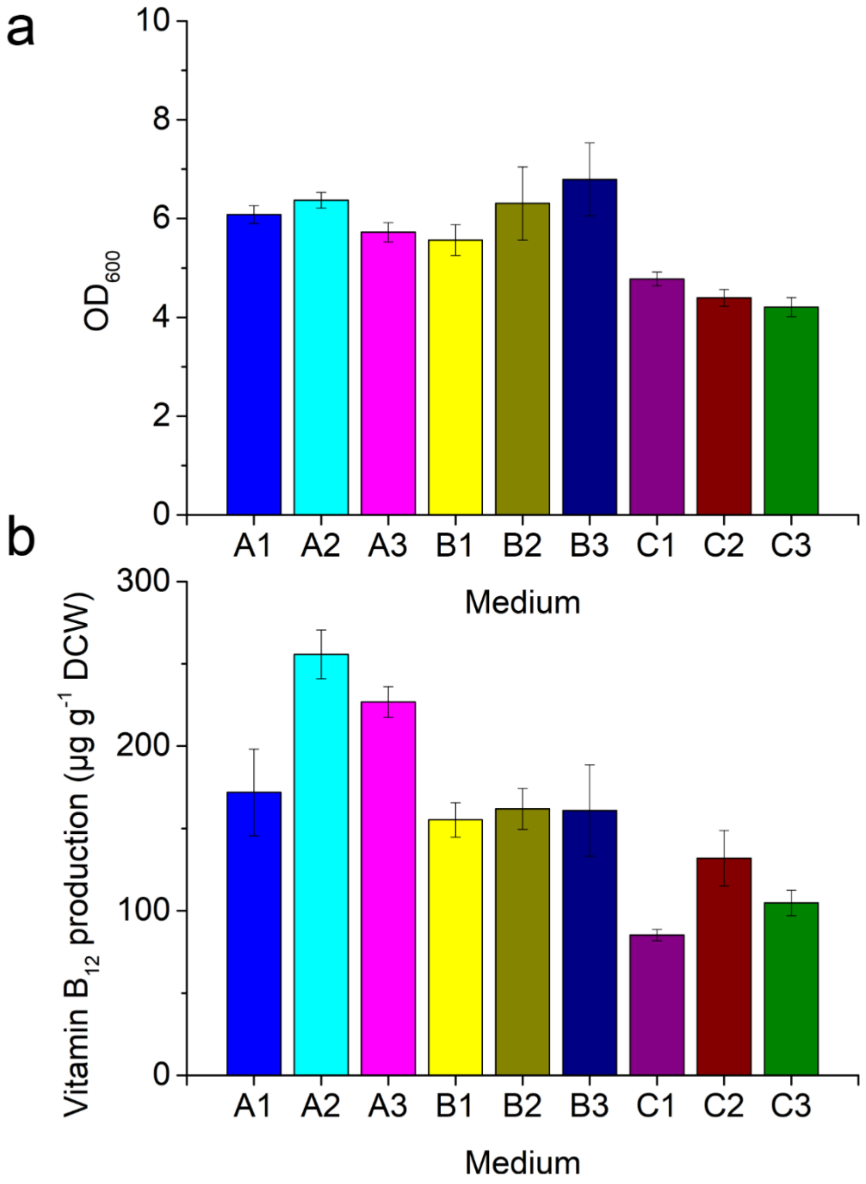

Supplementary Figure 16. Comparison of (**a**) the growth characteristics and (**b**) vitamin B_{12} productivity of FH364 cultured in various growth media. The composition of the medium is presented in detail in Supplementary Table 3. Error bars indicate standard deviations from triplicate biological replicates.

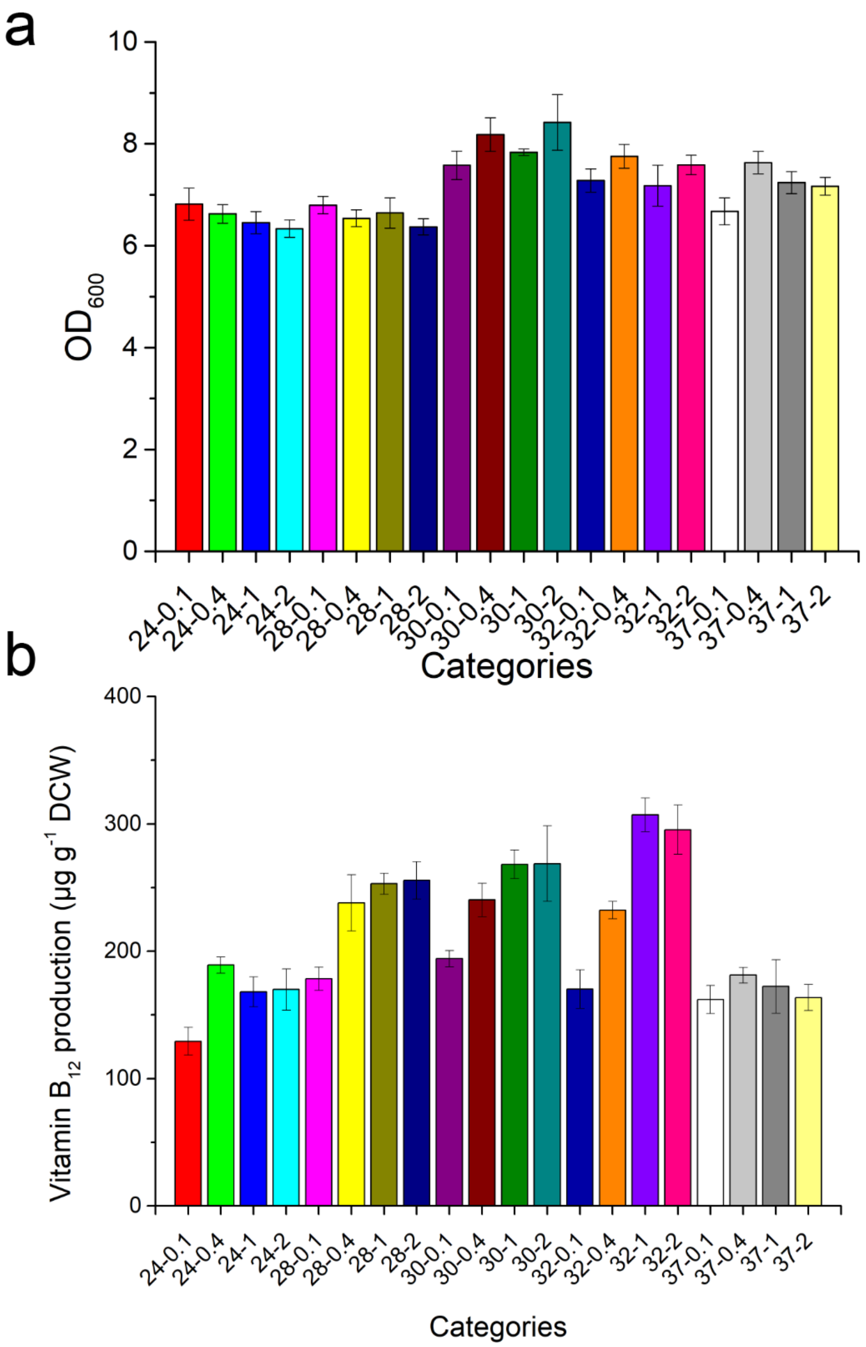

Supplementary Figure 17. Comparison of (**a**) the growth characteristics and (**b**) vitamin B_{12} productivity of FH364 under different conditions. FH364 was grown in CM-A2 medium for vitamin B_{12} production under different temperatures and with inducion by various IPTG concentrations. Error bars indicate standard deviations from triplicate biological replicates.

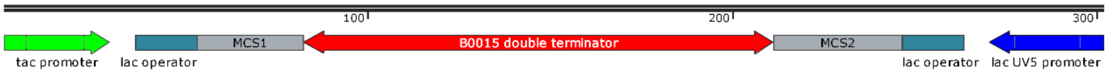

Supplementary Figure 18. Map of synthesized DNA fragment of the plasmid p15ASI. The fragment contains tac, lacUV5 promoters and a bidirectional terminator.

Supplementary Table 1. Effect of regulating copy numbers of plasmids on vitamin B_{12} production

Strains	Replicon of the plasmid containing Module 1	Replicon of plasmids containing Module 2	Replicon of plasmids containing Module 4	Vitamin B_{12} production
FH364	pMB1	CloDF13	p15A	171.81±26.38 μg g^{-1} DCW
FH378	pMB1	CloDF13	pSC101	139.56±5.77 μg g^{-1} DCW
FH380	pMB1	p15A	CloDF13	Not detected
FH381	pMB1	p15A	pSC101	Not detected
FH382	pMB1	pSC101	CloDF13	Not detected
FH383	pMB1	pSC101	p15A	Not detected

The copy numbers of pMB1, CloDF13, p15A, and pSC101 replicons are 20-30[2], ~300[3], 10-15[2], and 6-8[4], respectively. Error bars indicate standard deviations from triplicate (FH364) or duplicate (FH378, FH380, FH381, FH382, FH383) biological replicates.

Supplementary Table 2. Plasmid maintenance ratio of the recombinant *E. coli* strains

Strains	Time after IPTG induction (h)	Plasmid maintenance ratio (Kan^r)	Plasmid maintenance ratio (Str^r)	Plasmid maintenance ratio (Cm^r)	Plasmid maintenance ratio (Kan^r+ Str^r + Cm^r)
FH364	10	100 ± 0%	78 ± 7.89 %	100 ± 0%	68.84 ± 9.50%
	20	100 ± 0%	52.66 ± 7.17%	63.94 ± 6.25%	42.79 ± 4.30 %
FH378	10	100 ± 0%	72.78 ± 12.95%	77.22 ± 12.12%	66.67 ± 8.32%
	20	95 ± 3.27%	55.08 ± 7.24%	72.12 ± 9.09%	46.25 ± 5.28%
FH380	10	100 ± 0%	100 ± 0%	100 ± 0%	100 ± 0%
	20	81.98 ± 5.39%	83.14 ± 7.70%	100 ± 0%	70.35 ± 5.78%
FH381	10	100 ± 0%	100 ± 0%	100 ± 0%	100 ± 0%
	20	100 ± 0%	100 ± 0%	100 ± 0%	100 ± 0%
FH382	10	61.86 ± 13.14%	100 ± 0%	59.79 ± 9.91%	62.89 ± 23.11%
	20	58.34 ± 8.84%	62.50 ± 22.71%	48.34 ± 11.85%	40.9 ± 8.98%
FH383	10	100 ± 0%	100 ± 0%	100 ± 0%	100 ± 0%
	20	100 ± 0%	100 ± 0%	100 ± 0%	100 ± 0%

Error bars indicate standard deviations from duplicate biological replicates.

Supplementary Table 3. Optimization of media components to improve vitamin B_{12} production

Components	Concentrations								
Yeast extract	$5\ g\ l^{-1}$								
Tryptone	$10\ g\ l^{-1}$								
KH_2PO_4	$5\ g\ l^{-1}$								
$CoCl_2 \cdot 6H_2O$	$20\ mg\ l^{-1}$								
DMBI	$90\ mg\ l^{-1}$								
	A			B			C		
Glycine	$2\ g\ l^{-1}$			$1\ g\ l^{-1}$			$0.4\ g\ l^{-1}$		
Succinic acid	$10\ g\ l^{-1}$			$5\ g\ l^{-1}$			$2\ g\ l^{-1}$		
	A1	A2	A3	B1	B2	B3	C1	C2	C3
Betaine	$10\ g\ l^{-1}$	$5\ g\ l^{-1}$	$2\ g\ l^{-1}$	$10\ g\ l^{-1}$	$5\ g\ l^{-1}$	$2\ g\ l^{-1}$	$10\ g\ l^{-1}$	$5\ g\ l^{-1}$	$2\ g\ l^{-1}$

Supplementary Table 4. Optimization of growth temperatures and IPTG concentrations to improve vitamin B_{12} production

Categories		Growth temperature	IPTG concentrations
A	A1	24℃	0.1 mM
	A2		0.4 mM
	A3		1 mM
	A4		2 mM
B	B1	28℃	0.1 mM
	B2		0.4 mM
	B3		1 mM
	B4		2 mM
C	C1	30℃	0.1 mM
	C2		0.4 mM
	C3		1 mM
	C4		2 mM
D	D1	32℃	0.1 mM
	D2		0.4 mM
	D3		1 mM
	D4		2 mM
E	E1	37℃	0.1 mM
	E2		0.4 mM
	E3		1 mM
	E4		2 mM

Supplementary References

1. Deery E*, et al.* An enzyme-trap approach allows isolation of intermediates in cobalamin biosynthesis. *Nature chemical biology* **8**, 933-940 (2012).

2. Anthony JR, Anthony LC, Nowroozi F, Kwon G, Newman JD, Keasling JD. Optimization of the mevalonate-based isoprenoid biosynthetic pathway in Escherichia coli for production of the anti-malarial drug precursor amorpha-4,11-diene. *Metabolic engineering* **11**, 13-19 (2009).

3. Kang CW, Lim HG, Yang J, Noh MH, Seo SW, Jung GY. Synthetic auxotrophs for stable and tunable maintenance of plasmid copy number. *Metabolic engineering* **48**, 121-128 (2018).

4. Rong Fu W, Kushner SR. Construction of versatile low-copy-number vectors for cloning, sequencing and gene expression in Escherichia coli. *Gene* **100**, 195-199 (1991).
