# Supplementary material for: Metabolic engineering of Escherichia coli for de novo biosynthesis of vitamin B12
Source: Nat Commun. 2018 Nov 21;9:4917. doi: 10.1038/s41467-018-07412-6 (PMC6249242; doi:10.1038/s41467-018-07412-6)
Supplement: Supplementary file 5 — Description of Additional Supplementary Files [file 41467_2018_7412_MOESM5_ESM.docx]

Title: Supplementary Data 1.
Description: List of the strains and plasmids used in this study.

Title: Supplementary Data 2.
Description: Primers and guide RNA sequences used in this study.
